# Editorial: Cognate recognition, functional properties and immunotherapeutic applications of iNKT cells: leveling the road to the clinic

**DOI:** 10.3389/fimmu.2025.1552225

**Published:** 2025-01-23

**Authors:** A. Raul Castaño, Steven Anthony Porcelli

**Affiliations:** ^1^ Department of Cellular Biology, Physiology and Immunology, Autonomous University of Barcelona, Barcelona, Spain; ^2^ Department of Microbiology & Immunology, Albert Einstein College of Medicine, New York City, NY, United States

**Keywords:** iNKT cell, CD1d, CAR T cell, immunotherapy, glycolipid antigen, alpha-galactosylceramide

This coming year marks the 30^th^ anniversary of the connection between a distinctive subset of T cells expressing the NK cell associated marker NK1.1 and the emerging area of lipid and glycolipid antigen presentation by CD1 molecules (1). This connection was established by the discovery that these unusual T cells, now referred to as invariant NKT cells (iNKT cells), were activated by recognition of cells expressing CD1d, a member of the CD1 family of lipid antigen presenting molecules (2–5). Shortly later, the prototypical agonist presented by CD1d to iNKT cells, α-galactosyl ceramide (αGC) was characterized (6). The demonstration of the stimulation of potent antitumor responses by αGC (7) opened a prolific field of basic and applied research, establishing the immunotherapeutic potential of iNKT cells for cancer and other diseases (8). However, to this date subsequent clinical trials have not yet yielded clear success. This may reflect a variety of complex issues related to subtle but important features of iNKT cell activation, differentiation and specificity that remain to be fully explored, thus hindering the development of effective iNKT cell-based therapies.

The strong capacity of iNKT cells to secondarily transactivate other immune cells, and to polarize the immune response towards different functional outcomes has been clearly defined. However, an integrated understanding of iNKT cell activation, the molecular interactions governing agonist presentation and recognition, and how these translate into different outcome remain incomplete. In addition, comprehensive understanding of the subtle differences between human and mouse iNKT cells, important for preclinical modeling of potential therapies, is lacking. The reviews assembled in this Research Topic discuss some of these issues and highlight new immunotherapeutic applications that may be at the forefront of clinical implementation in coming years.

An example of the complexities regarding involvement of iNKT cells in disease states is provided by their conflicting roles in allergic asthma, as thoroughly reviewed by Gutierrez-Vera et al. From animal models to studies in patients, controversial and contradictory results illustrate the multifaceted functions of iNKT cells, the misleading conclusions from some overused animal models, the influence of microbiota and genetic background, and the difficulties involved with translation of animal results to humans. Potential therapeutic interventions including secondary activation of regulatory T and B cells, specific activation of an iNKT10 subset or the use of analogs deviating immune response away from the characteristic inflammatory Th2 response, are proposed, but still far from being attainable.

Cognate activation of iNKT cells by recognition of glycolipids translates into distinct functional outcomes upon recognition of subtle structural differences. Praveena and Le Nours show the extensive structural studies, including 3D structures of the trimolecular invariant TCR-glycolipid-CD1d complex, performed on multiple different agonists to understand the rules that may allow precise control of the subsequent immune response. Compounds modified at either the sugar head group or the lipid tails that improve stability by increasing interaction with the TCR or with CD1d cause polarization towards Th1 responses, although the precise rules which determine the final *in vivo* outcome are not yet fully grasped. For instance, ligands with similar TCR affinities and interface structure can induce different responses in straightforward cellular assays (9). Furthermore, cellular functional assays do not necessarily anticipate *in vivo* responses as weak agonists may in some cases control tumor growth as efficiently as strongly agonistic αGC (10). Thus, considerable gaps remain in our ability to link structural determinations of iNKT cell ligand recognition with the functional outcome.

CAR-T cells are effective medical treatment for certain hematopoietic cancers, but there are many challenges currently to extending their use, particularly for solid tumors. As a significant improvement, iNKT cells are proposed as an ideal platform for building effective cellular therapy. O´Neal et al. review preclinical and clinical trials on the use of iNKT cells in this regard for fighting malignant diseases, as suggested by some of their intrinsic properties including their intrinsic off-the-shelf nature, potent transactivation of other anti-tumor cells, superior ability to effectively infiltrate the tumor microenvironment, dual targeting of CD1d+ tumors and the CAR antigenic specificity, superior safety profile and resistance to allogeneic rejection (11).

Critical limitations imposed by the scarcity of iNKT cells are close to being solved, as procedures to safely obtain large quantities of cells are now being established (12). However, some discrepancies with animal models have emerged. Equal or superior competence of human CD4^+^ iNKT-CAR cells contradict the previously reported functional capacity of CD4^-^ subsets to induce Th1 responses with the highest tumor eradication potential (13). Some of the discrepancies may arise from differences in iNKT cell development and subpopulations between mice and humans, discussed by Pellici et al. The clearly characterized murine subsets defined by expression markers, transcription factors and functional outcomes generated during thymic maturation (iNKT1, iNKT2, iNKT17 and others), are less clearly demarcated in humans. The iNKT cell maturation process in humans takes place mostly in the periphery, where CD4^+^CD161^-^ immature thymic iNKT cells mostly become CD4^-^CD161^+/-^, without such clearly polarized functions. This suggests that CAR-iNKT cell efficacy and precision of therapeutics may be improved by incorporating expression of cytokines such as IL-15, IL-7 or IL-21.

Another promising application derives from their regulatory properties, as iNKT cells prevent development of graft versus host disease (GVHD) (O´Neal et al.) in hematopoietic stem cell transplants (HSCT) (14). Adoptive transfer of low numbers of iNKT cells prevented GVHD in allogeneic settings in mice, without inhibiting anti-leukemia (GVL) efficacy. This is associated with induction of Tregs by iNKT2 and iNKT17 subsets (15). Similarly, in human HSCT grafts the reduction of GVHD correlates with increased iNKT cell numbers. Xenogeneic humanized models also demonstrated GVHD suppression, surprisingly attributed to the CD4^-^ subset. Again, subset differences between species or particularities of the immunocompromised models studied are potentially important factors.

Overall, despite the many encouraging findings, extensive research on human iNKT cell subsets and functional differences to reconcile results in preclinical animal models vs. human settings must be addressed to pave the road toward effective iNKT cell-based therapies. A more thorough understanding of complexities involved in agonist recognition and iNKT response, along with generation of animal models that more closely recapitulate human responses (16) to rigorously assess the potential of various iNKT cell directed therapeutic approaches, including CAR constructs, should be major goals for closing the present gap in therapeutic potential that remains between preclinical and clinical studies.
